# Pathological Relevance of Anti-Hsp70 IgG Autoantibodies in Epidermolysis Bullosa Acquisita

**DOI:** 10.3389/fimmu.2022.877958

**Published:** 2022-04-20

**Authors:** Stefan Tukaj, Jagoda Mantej, Krzysztof Sitko, Detlef Zillikens, Ralf J. Ludwig, Katja Bieber, Michael Kasperkiewicz

**Affiliations:** ^1^ Department of Molecular Biology, Faculty of Biology, University of Gdańsk, Gdańsk, Poland; ^2^ Department of Dermatology and Center for Research on Inflammation of the Skin, University of Lübeck, Lübeck, Germany; ^3^ Lübeck Institute of Experimental Dermatology and Center for Research on Inflammation of the Skin, University of Lübeck, Lübeck, Germany; ^4^ Department of Dermatology, Keck School of Medicine, University of Southern California, Los Angeles, CA, United States

**Keywords:** autoimmune bullous diseases (AIBDs), epidermolysis bullosa acquisita (EBA), heat shock proteins (Hsps), Hsp70, autoantibodies, nuclear factor kappa B (NF-κB), interferon gamma (IFN-γ)

## Abstract

Stress-induced heat shock protein 70 (Hsp70) is a key intra- and extracellular molecular chaperone implicated in autoimmune processes. Highly immunogenic extracellular Hsp70 can activate innate and acquired (adaptive) immune responses driving the generation of anti-Hsp70 autoantibodies that are frequently observed in inflammatory/autoimmune disorders. We recently described the direct pathological role of extracellular Hsp70 in epidermolysis bullosa acquisita (EBA), an anti-type VII collagen autoantibody-mediated autoimmune blistering skin disease. Here, we determined the role of anti-Hsp70 autoantibodies in EBA. We observed that circulating anti-Hsp70 IgG autoantibodies were significantly elevated in EBA patients compared to healthy individuals and positively correlated with serum levels of pro-inflammatory interferon gamma (IFN-γ). The pathophysiological relevance of anti-Hsp70 IgG autoantibodies was demonstrated in an antibody transfer-induced EBA mouse model in which elevated serum levels of anti-Hsp70 IgG were found. In addition, anti-Hsp70 IgG-treated animals had a more intense clinical and histological disease activity, as well as upregulated nuclear factor kappa B (NF-κB) activation in skin biopsies compared to isotype-treated animals. Our results suggest that autoantibodies to Hsp70 may contribute to EBA development *via* enhanced neutrophil infiltration to the skin and activation of the NF-κB signaling pathway in an IFN-γ-associated manner.

## Introduction

Cell-protecting inducible heat shock protein 70 (Hsp70) is a highly conserved molecular chaperone involved in intracellular protein folding and homeostasis. Hsp70 can be up-regulated by multiple stress stimuli and released to extracellular compartments, where it influences both innate and adaptive immune cells (1). Passively or actively released to the extracellular milieu, Hsp70 may drive the generation of circulating anti-Hsp70 autoantibodies. In fact, increased serum levels of these autoantibodies were recorded in some autoimmune diseases including dermatitis herpetiformis (2), coeliac disease (3), and rheumatoid arthritis (4). In addition, our recent studies revealed that extracellular autologous Hsp70 represents a pathophysiological factor in epidermolysis bullosa acquisita (EBA) (5). EBA is one of the less common autoimmune blistering skin diseases characterized by the presence of autoantibodies to type VII collagen (COL7) of the cutaneous basal membrane zone. In experimental models of inflammatory EBA, anti-COL7 IgG binding is followed by reactive oxygen species (ROS) and matrix metalloproteinases (MMP) producing neutrophils, resulting in proteolytic degradation of the dermal-epidermal junction and blister formation (6).

This study aimed to determine the role of circulating anti-Hsp70 autoantibodies in a cohort of patients with EBA and healthy controls. Their presumed pathological effects were confirmed in a well-established experimental mouse model of EBA.

## Materials and Methods

### Human Blood Samples

Sera from healthy donors (n=40) and age-matched patients with active EBA (n=20) were used in this study. EBA patients were included fulfilling the following criteria: skin lesions resembling EBA (inflammatory subtype, n=10; unspecified subtype, n=10), linear IgG and/or IgA staining at the skin basal membrane zone by direct immunofluorescence microscopy and circulating anti-COL7 IgG autoantibodies by both indirect immunofluorescence microscopy and immunoblotting. The use of sera was approved by the Ethics Committee of the University of Lübeck (Germany), and written informed consent was obtained in accordance with the Declaration of Helsinki.

### Analysis of Circulating Anti-Hsp70 Immunoglobulins

Levels of circulating anti-Hsp70 Ig were evaluated by enzyme-linked immunosorbent assay (ELISA) as shown previously (4, 5).

### Serum INF-γ Measurements

Serum levels of mouse and human IFN-γ were measured by commercially available ELISA kits (BioLegend and MyBiosource, respectively).

### Generation of the vWFA2 Recombinant Protein and Anti-Murine vWFA2 IgG

Recombinant murine vWFA2 of COL7 was generated as described previously (7). Rabbit anti-murine vWFA2 IgG (anti-COL7 IgG) was produced and isolated as described previously (8). Immunoreactivity of IgG fractions to COL7 was examined by immunofluorescence microscopy on mouse skin.

### Disease Induction

♀ BALB/c mice (6 weeks of age) were purchased from the Tri-City University Animal Facility — Research Service Center (Poland). Induction of EBA by repetitive anti-COL7 IgG transfer in mice followed published protocols (5, 9). Disease activity was expressed as the percentage of body surface area covered by skin lesions and determined at three time points as described previously (5, 9).

### Treatment of Mice

Naïve mice were injected (50 μg per mouse) with a single intraperitoneal injection of mouse anti-Hsp70 IgG_1_ monoclonal antibodies (clone BRM-22; Sigma) or IgG_1_ isotype control (Sigma) one day before the initial anti-COL7 IgG injection.

### Histopathology

Mouse ear skin samples were fixed in 4% buffered formalin and embedded in paraffin. 6 μm tissue sections were stained with hematoxylin and eosin (H&E). Dermal neutrophil infiltration was scored blindly by an independent researcher on a scale from 0 to 4, i.e., 0, none; 1, slight; 2, moderate; 3, marked; and 4, very marked as described previously (5).

### NF-kB p65 Activity

NF-κB p65 activity was measured in nuclear extracts of the skin by a NF-kB p65 Transcription Factor Assay Kit following the manufacturer’s instruction (Abcam).

### Flow Cytometry

Whole blood cells were labeled with anti-Ly6G-FITC (BioLegend) and anti-CD62L-APC (BioLegend) in CyLyseTM reagents (Sysmex). Stained viable granulocytes were analyzed with a flow cytometer (CyFlow Cube 6, Sysmex).

### H_2_O_2_ Measurement

Hydrogen peroxide (H_2_O_2_) levels were assayed using the Amplex^®^UltraRed fluorochrome (Molecular Probes) and Varioskan Reader (Thermo Fisher Scientific) as described previously (5).

### Western Blotting

Matrix metalloproteinase 9 (MMP-9) or neutrophil elastase (NE) protein expression was analyzed by immunoblotting as described previously (5). Briefly, ear skin lysates were separated in polyacrylamide gel under denaturing conditions (SDS-PAGE) and transferred onto nitrocellulose membrane (Bio-Rad). The membrane was incubated in blocking buffer (TBS) containing 3% non-fat milk, followed by incubation with antibodies to MMP-9 (1:1000; Abcam), NE (1:1000; Cell Signaling Technology) or β-actin (1:1000; Cell Signaling Technology) at room temperature. HRP-coupled goat anti-mouse or anti-rabbit secondary antibodies (1:2000; BioLegend or 1:2000; Sigma-Aldrich, respectively) were used. Amersham ECL Plus Western Blotting Detection Reagents (GE Healthcare) were used to visualize the reaction. Protein levels relative to β-actin were measured by densitometry (Image Lab 6.1.0).

### Statistical Analyses

Statistical calculations were carried out using GraphPad Prism 9 (San Diego, CA). To verify whether the data had normal distribution, the Shapiro-Wilk test was used. Data was analyzed by Student’s t test, Mann–Whitney U test, or Spearman’s rank correlation test, and p values less than 0.05 were considered significant.

## Results

### Levels of Circulating Anti-Hsp70 IgG Autoantibodies Are Increased in Patients With EBA

**Figure 1:**
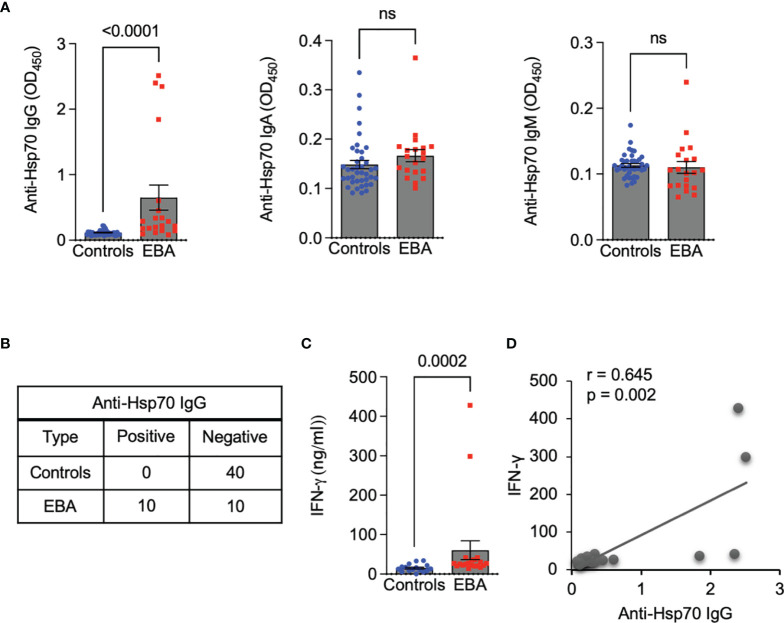
Circulating anti-Hsp70 IgG autoantibodies are elevated in patients with EBA and positively correlated with serum levels of pro-inflammatory IFN-γ. **(A)** Levels of anti-Hsp70 IgG, IgA, and IgM autoantibodies in sera of EBA patients (n = 20) and age-matched healthy individuals (n = 40), measured by ELISA. **(B)** Contingency analysis showing anti-Hsp70 IgG positivity in 50% of EBA patients but not in healthy controls. **(C)** Levels of IFN-γ in sera of EBA patients (n = 20) and healthy controls (n = 16), measured by ELISA. **(D)** Analysis of a correlation between serum levels of anti-Hsp70 IgG and IFN-γ in EBA patients. ns, not significant.

We observed that serum levels of anti-Hsp70 IgG were significantly higher in EBA patients (n = 20) when compared to age-matched healthy individuals (n = 40) (**Figure 1A**), which was also true for a subgroup analysis with EBA patients for whom information about the clinical variant was available (i.e., inflammatory type, n=10) (0.21 ± 0.09 vs. 0.11 ± 0.03, respectively; p=0.0019). In contrast, levels of anti-Hsp70 IgA and IgM were similar between both groups (**Figure 1A**). Cut-off value calculated as 3 x standard deviation (SD) above the mean of the controls revealed that 50% of EBA patients were anti-Hsp70 IgG positive, whereas none of the healthy controls displayed such positivity (**Figure 1B**). In addition, significantly elevated serum levels of IFN-γ in EBA patients as compared to controls (**Figure 1C**) were positively correlated with serum levels of anti-Hsp70 IgG (**Figure 1D**).

### Treatment With Anti-Hsp70 Antibodies Boosts Induction of Experimental EBA

**Figure 2:**
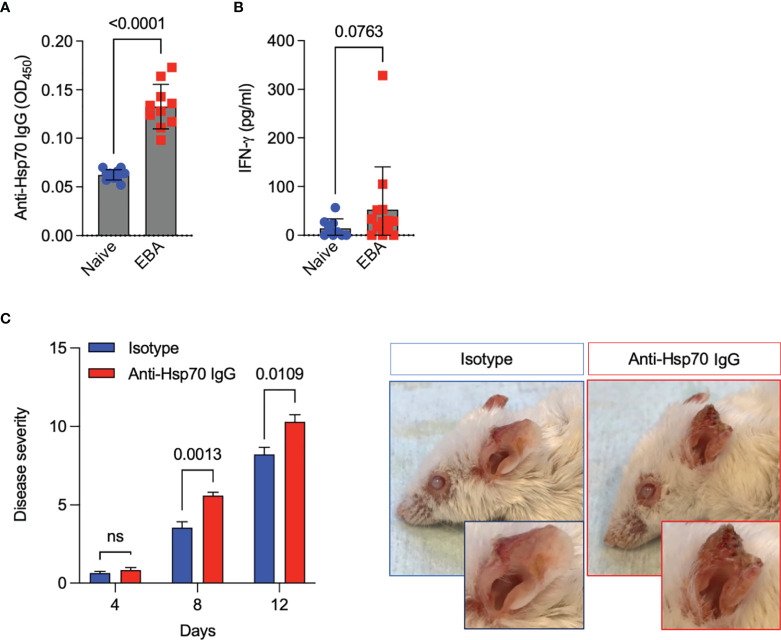
Anti-Hsp70 antibody treatment aggravates experimental EBA. **(A)** Blood levels of anti-Hsp70 IgG and **(B)** IFN-γ in naïve mice and mice with induced EBA were measured by ELISA at day 12. The results are expressed as mean values ± SEM of 9-13 mice per group. **(C)** Naïve BALB/c mice were injected intraperitoneally with mouse anti-Hsp70 IgG or IgG isotype control one day before the initial anti-COL7 IgG injection. Clinical scores were calculated as the percentage of the body surface area covered by EBA lesions. Representative clinical pictures of isotype control- and anti-Hsp70 IgG-treated mice at the end of the observation period (day 12) are shown on the right. The results are expressed as mean values ± SEM of 5 mice per group ns, not significant.

To validate the role of anti-Hsp70 IgG antibodies *in vivo*, the antibody transfer-induced EBA animal model was used. First, we observed that induction of experimental EBA was paralleled by both the generation of circulating anti-Hsp70 IgG (**Figure 2A**) and a trend towards higher blood levels of IFN-γ as compared to naïve mice (**Figure 2B**). Secondly, in a separate experimental setting, we found that naïve mice injected intraperitoneally with murine anti-Hsp70 IgG one day before the initial anti-COL7 IgG injection had a significantly higher clinical disease activity when compared to isotype-treated EBA mice (**Figure 2C**).

### Anti-Hsp70 IgG Antibody Treatment Leads to Pronounced Dermal Neutrophil Infiltration and NF-κB Activity in Experimental EBA

**Figure 3:**
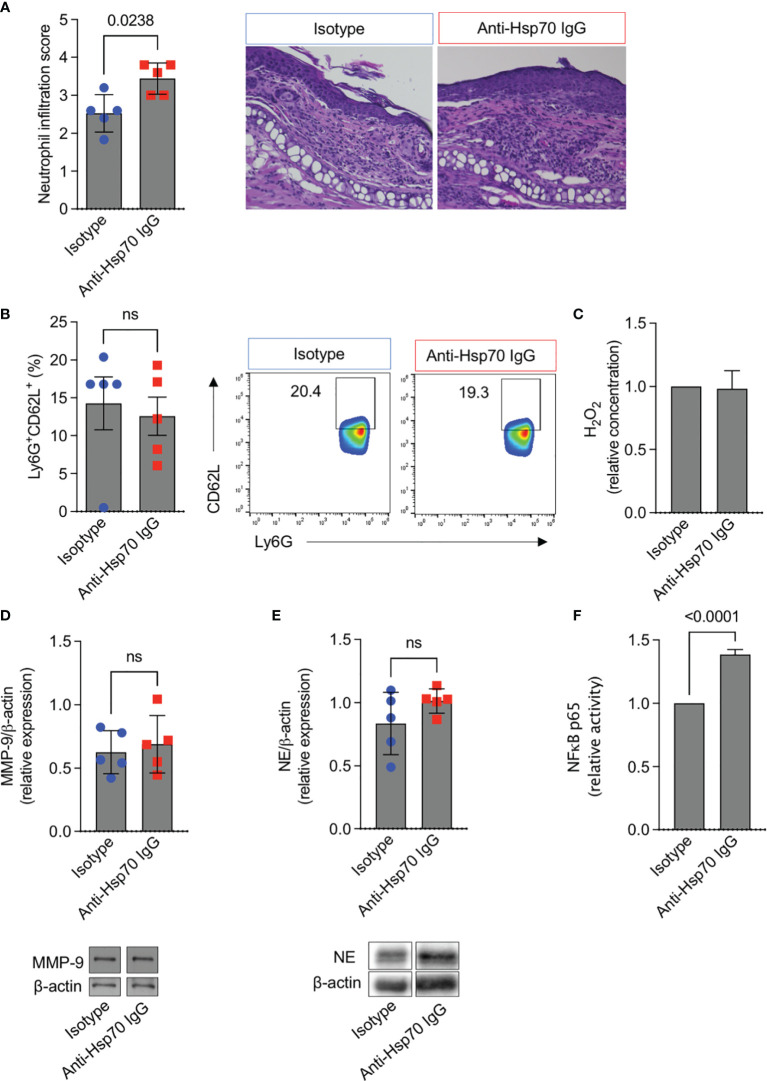
Anti-Hsp70 IgG antibody treatment leads to enhanced dermal neutrophil infiltration and NF-κB activity in mice with EBA. Naïve BALB/c mice were injected intraperitoneally with murine anti-Hsp70 IgG or IgG isotype control one day before the initial anti-COL7 IgG injection. **(A)** Dermal neutrophil infiltration scores of H&E-stained skin sections at the end of the observation period (day 12). Representative histological images are shown on the right. **(B)** Flow cytometric analysis of peripheral blood neutrophil activity (Ly6G^+^CD62L^+^) at the end of the observation period (day 12). Representative cytometric results are shown on the right. **(C)** Plasma H_2_O_2_ levels at the end of the observation period (day 12). Cutaneous expression of **(D)** MMP-9 or **(E)** neutrophil elastase (NE) at day 12. Corresponding representative western blot (below) with MMP-9 or NE expression relative to β-actin levels using densitometry measurements. **(F)** Relative NF-κB p65 activity was analyzed in nuclear extracts of the skin by ELISA at the end of the observation period (day 12). Data are expressed as mean ( ± SEM) of 5 mice per group. ns, not significant.

To further evaluate the mechanism of anti-Hsp70 IgG action in EBA development, skin infiltration and the activity of neutrophils, the key effector cells in experimental EBA (6), were studied. In addition, the activation of nuclear factor kappa B (NF-κB), which is known to play a key role in autoimmunity and inflammation (10), was monitored. We found that the dermal infiltration by neutrophils was significantly higher in anti-Hsp70 IgG-treated mice as compared to the isotype control-treated group (**Figure 3A**), although no intra-individual association between serum anti-Hsp70 IgG and dermal neutrophil infiltration was recorded in untreated EBA mice (data not shown). While circulating neutrophil activity (Ly6G^+^CD62L^+^) (**Figure 3B**), plasma H_2_O_2_ levels (**Figure 3C**), and skin expression of MMP-9 (**Figure 3D**) or neutrophil elastase (**Figure 3E**) were not affected by anti-Hsp70 IgG antibody treatment, NF-κB activation was significantly enhanced in lesional skin biopsies of EBA mice treated with the anti-Hsp70 IgG antibody compared to isotype-injected EBA mice (**Figure 3F**).

## Discussion

Hsp70 is a highly conserved molecular chaperone involved in intracellular protein folding and cellular homeostasis. Living or dead cells can actively or passively release Hsp70 to the extracellular space, respectively, where it influences both innate and adaptive immune cells. The role of extracellular Hsp70 in autoimmunity, however, is still enigmatic due to conflicting outcomes regarding its contribution to the development or maintenance of pathological conditions, depending on type of diseases, experimental conditions, and the general cellular niche (1, 5, 11). For instance, Hsp70 treatment suppressed psoriasis and autoimmune arthritis in preclinical models (11, 12). On the other hand, extracellular Hsp70 has an inflammation-promoting role in EBA (5). In more detail, circulating levels of Hsp70 were significantly elevated in mice with experimental EBA as compared to naïve mice, and Hsp70-treated EBA mice had a more intense clinical disease severity compared to untreated EBA mice (5). Since naturally occurring circulating anti-Hsp70 autoantibodies were found to be elevated in several autoimmune diseases (13), we aimed to define the role of anti-Hsp70 antibodies in EBA.

This research involved both EBA patients and the EBA animal model which reflects the inflammatory type (vs. mechanobullous type) of human EBA. Here, we observed that IgG autoantibodies against Hsp70 were significantly elevated in the serum of patients with EBA, including those with the inflammatory variant, when compared to healthy individuals. This observation is in line with what we have previously described in patients with another autoimmune blistering disease, i.e., dermatitis herpetiformis (2). In contrast, patients suffering from bullous pemphigoid or pemphigus vulgaris, the most common forms of autoimmune bullous dermatoses, had unchanged serum levels of anti-Hsp70 IgG as compared to healthy controls (2). This discrepancy may suggest disease subtype-specific humoral autoreactivity to Hsp70 within the same group of autoimmune disorders. In this context, potential differences between the inflammatory and mechanobullous variants of EBA remain in need of further elucidation. Likewise, the mechanism of anti-Hsp70 IgG production in EBA patients is still not fully understood but may possibly be linked to skin tissue damage-related release of Hsp70 to the extracellular space with consecutive activation of the humoral immune response. An indirect pathophysiological significance of anti-Hsp70 IgG in EBA patients may be evidenced by the positive correlation between serum levels of anti-Hsp70 IgG and IFN-γ, the latter known to be implicated in EBA (14–16). Since a positive correlation between circulating anti-Hsp90 IgG and IFN-ɣ has been previously also found in patients with rheumatoid arthritis (4), it is likely that anti-Hsp autoantibodies are involved in the development of some autoimmune diseases in an IFN-ɣ-associated manner.

The pathophysiological relevance of anti-Hsp70 antibodies was experimentally demonstrated in the murine model of EBA. In this well-established antibody transfer-induced EBA model, anti-COL7 antibodies lead to neutrophil infiltration into the skin which directly causes subepidermal blister formation by ROS- and MMP-mediated disruption of adhesion molecules (6). Here, in line with EBA patients, we observed that induction of experimental EBA was paralleled by both the generation of circulating anti-Hsp70 IgG and a trend towards higher blood levels of IFN-γ as compared to healthy naïve mice. In addition, we demonstrated that anti-Hsp70 IgG-treated mice had a more intense clinical and histological disease activity of EBA compared to the control group. Mechanistically, an increase of NF-κB activation in the skin of anti-Hsp70 IgG-treated mice with EBA was found. Since the NF-κB signaling pathway is known to play a key role in inflammatory processes, it may represent a possible target for the treatment of autoimmune diseases including those of the bullous type (10, 17–19).

## Summary

In summary, based on serological studies using EBA patients and preclinical observations, we postulate that autoantibodies to Hsp70 may contribute to EBA development *via* enhanced neutrophil infiltration to the skin and activation of the NF-κB signaling pathway in an IFN-γ-associated manner. This work expands existing knowledge about the direct contribution of extracellular Hsp70 in EBA, suggesting a synergistic interplay between Hsp70 *per se* and the respective autoantibody response in the pathophysiology of EBA which may also have implications for novel biomarker development.

## Data Availability Statement

The original contributions presented in the study are included in the article/supplementary material. Further inquiries can be directed to the corresponding author.

## Ethics Statement

The studies involving human participants were reviewed and approved by Ethics committee of the University of Lübeck (Germany). The patients/participants provided their written informed consent to participate in this study. The animal study was reviewed and approved by local authorities of the Animal Care and Use Committee (Bydgoszcz, Poland).

## Author Contributions

Study design and conceptualization: ST. Supervision: ST. Analysis: ST, JM, KS, and KB; Original Draft Preparation: ST and MK. Data interpretation and critical revision of the manuscript: all authors. All authors contributed to the article and approved the submitted version.

## Funding

This study was supported by the Polish National Science Centre (NCN), grant no. 2017/25/B/NZ6/00305 and 2020/39/B/NZ6/00357 and by structural funding by Excellence Cluster 2167 Precision Medicine in Chronic Inflammation (Deutsche Forschungsgemeinschaft).

## Conflict of Interest

The authors declare that the research was conducted in the absence of any commercial or financial relationships that could be construed as a potential conflict of interest.

## Publisher’s Note

All claims expressed in this article are solely those of the authors and do not necessarily represent those of their affiliated organizations, or those of the publisher, the editors and the reviewers. Any product that may be evaluated in this article, or claim that may be made by its manufacturer, is not guaranteed or endorsed by the publisher.
